# Tackling Obesity: A Qualitative Exploration of a Whole School Food Policy Guideline

**DOI:** 10.7759/cureus.105629

**Published:** 2026-03-21

**Authors:** Eve Kerins, Hannah Opstad

**Affiliations:** 1 Paediatrics and Child Health, University College London, London, GBR; 2 Haematology, Guy's Hospital, London, GBR; 3 Paediatrics, Haringey Council, London, GBR; 4 Paediatrics, St George's Hospital, London, GBR

**Keywords:** childhood obesity, children, children nutrition, food policy guidelines, healthy eating, local council, obesity and overweight, schools, water-only schools, whole school approach

## Abstract

Introduction

Childhood obesity is an ever-increasing public health concern in England. Schools offer a unique opportunity to influence childhood obesity. Children spend a significant proportion of their time at school, and there is an opportunity to equip them with lifelong knowledge and skills whilst they are highly influenced by their environment. This Whole School Food Policy Guideline was designed to help schools make whole system changes to their culture and ethos, promoting healthier eating and life choices for their pupils. This project disseminated the guidelines to all schools in the London Borough of Haringey through the local authority's Public Health team, and its impact was explored. The aim of this project was to develop a cost-effective resource, the Whole School Food Policy Guideline, to assist schools in creating environments that promote healthy life choices.

Methods

Development involved collaboration with local dietitians, teachers and chefs to combine ideas and resources surrounding healthy school food policies. Draft guidelines were reviewed by all contributors. Once finalised, guidelines were printed into booklets and sent to all Haringey schools in February 2020. Semi-structured telephone interviews were conducted in April 2020 with "Healthy School Leads (HSL)" from three local schools: one primary, one secondary and one special educational needs (SEN) school. The aim was to assess initial impressions of the guideline and its perceived usefulness. The data were thematically analysed, and the resulting themes were validated by both researchers.

Results

Five main themes emerged during analysis, labelled: "utility", "facilitators", "barriers", "special schools" and "guideline improvements and other resources". The guideline was reported as a useful resource with appropriate suggestions for change. This included being adaptable to the needs of the children with SEN. It was particularly valued as a council-produced document, adding a layer of support when implementing change. Resistance to change from parents, children and staff was reported as a previously encountered barrier to healthy change, and the guidelines' informative tone and authority from professionals were useful to address this. The packed lunch guide was well-received by all interviewees, directly addressing their concerns about the quality of food brought from home. The only implemented change at the point of data collection was that this guide had been sent by one school to parents via email. A common view was the feasibility of the recommended water-only policy. All schools mentioned this initiative; one interviewee felt their existing water-only policy needed to be “tightened up”, and both other schools considered introducing this idea. It was found that water-only policies are an easily accepted change in comparison to the emotive and controversial nature of food-based policies, suggesting this change would be easily established.

Conclusion

The guideline fills a gap in supporting schools implementing changes as a Whole School Approach to improve students' health. Its potential impact depends on integration within current practice. The findings from this study suggest that future efforts should focus on water-only policies, working collaboratively with parents and continuing to engage passionate staff who drive positive, meaningful change within their school.

## Introduction

Trends in childhood obesity across England are monitored through the National Child Measurement Programme (NCMP), which annually measures the body mass index of Reception and Year 6 students across all mainstream state-maintained schools. In 2024-2025, over one in ten children start primary school obese, increasing to one in five leaving primary school [1]. Omitting the peak during the COVID-19 pandemic, the rate of childhood obesity continues to rise year on year [1]. Childhood obesity is likely to persist into adulthood, alongside increased susceptibility to potentially life-threatening non-communicable conditions, namely diabetes, cardiovascular disease and some cancers [2]. This comes with an immense associated cost to the NHS, with approximately £6.5 billion per year on overweight- and obese-related ill-health [3]. There is an urgent need to address this problem.

Schools offer a unique opportunity to influence childhood obesity. This is recognised by the World Health Organisation. Their report on ending childhood obesity emphasised the importance of schools in creating a health-promoting environment and improving nutrition literacy by incorporating this into the core curriculum [4]. UK children spend just over half the year in school [5], and particularly those under the age of eight, are highly influenced by their environment [6].

Healthy eating policies in schools have been shown to effectively improve dietary behaviour [7]. Current research shows that school-based obesity prevention interventions should take a "Whole School Approach", where a healthy lifestyle is integral to the school’s ethos, policies, curriculum and environment [8].

Location

This project was based in the North London Borough of Haringey in 2019-2020. In the preceding academic year, 23% of children starting primary school were overweight or obese, rising to 38.4% of children in Year 6 [9]. In Reception, the rate of overweight and obesity was only 0.4% higher than the rest of England, but was 4.1% higher by Year 6.

Haringey is the fourth most deprived London borough [10]. There is a socioeconomic divide across the borough, with higher levels of deprivation in the east [10]. This influences obesity rates, with children in the east more likely to be overweight or obese than their western counterparts [11]. Children from minority ethnic groups are also more likely to be overweight or obese. This echoes findings by the NCMP, which has identified that local and regional variations in rates of childhood obesity are driven by inequalities in deprivation and ethnic groups [1].

Haringey Council is taking a Whole Systems Approach to tackling obesity, aiming to create an environment that promotes healthy behaviour change. Part of this focuses on schools; Haringey Council’s Healthy Schools awards support schools to improve student health and wellbeing [12]. Previously, there has been a focus on encouraging physical activity through the Daily Mile, where students walk or run for 15 minutes daily [13]. Schools have a member of staff who coordinates their healthy approach, known as the Healthy School Lead (HSL).

This article was previously presented as a poster and abstract at the Royal College of Paediatrics and Child Health Conference in June 2022.

## Materials and methods

Guideline development and distribution

This project developed a Whole School Food Policy Guideline, a free resource sent to all local schools in the borough by the council. It recommends healthy changes to develop a whole school food policy and includes a packed lunch guide for parents. Development involved collaboration with multiple professionals with relevant expertise and experience.

The guideline outlines the borough’s NCMP statistics. It illustrates the scale of the problem using colourful pie charts and people graphs. It describes the negative impact of childhood obesity using simple language and clearly explains that schools hold the potential to make an enormous impact on a child’s life.

Local dietitians reviewed the National School Food Standards and created an enhanced, healthier version for Haringey. This provides clear guidance on the quantity of different types of food that should be provided per week. The categories are starchy foods, protein, fruit and vegetables, milk and dairy, healthier drinks and foods high in fat, sugar and salt. Recommended portion sizes for different age groups were also provided, which were photographed to provide a visual aid. The printable “Packed lunch guide for parents” included a “pick and mix” tool for the different food categories, and clear guidance on how to provide a balanced meal.

The guideline also includes guidance for schools when procuring external caterers. It recommends questions to ask during the procurement process regarding topics such as portion sizing, supply chains and processed foods. It lists the expectations a school should have of a caterer, for example, “ideally, the produce will be fresh, local and seasonal” and “they will provide balanced meals with appropriate portion sizes”.

Teachers and chefs from other schools with successful healthy initiatives contributed their ideas to case studies. This included schools with weekly cooking sessions, compostable with no plastic waste snack policies, and competitive token reward schemes for healthy meals. It also details existing initiatives focused on improving school food policies, which the schools could get involved in.

Other recommendations include improving the school’s food culture and ethos, with ideas such as Teacher Tuesdays, where the teachers eat with the children once a week, or the older students being given responsibilities and acting as waiters during mealtimes. There’s advice on improving the quality of the tuck shop, and providing positive “nudges” where healthier options are easier to choose, such as having healthy snacks within easy reach and smaller plates making smaller portions appear bigger. The guideline also provides information on School Nutrition Action Groups, where representatives from across the school who have an interest in the school food meet to review the existing food policy, find areas to be improved and develop appropriate action plans. There is also advice on supporting children who are “fussy eaters”.

The guideline provides advice on incorporating education around food into a school’s existing practice. Four lesson plans were included, where healthy eating ideas were incorporated into the existing National Curriculum content. It also provides guidance on developing a teaching kitchen and food-growing spaces.

Guideline drafts were reviewed by councillors and all contributors, who validated their additions and provided feedback. The printed guidelines were sent to all schools in February 2020, with interviews held the following April.

**Figure 1:**
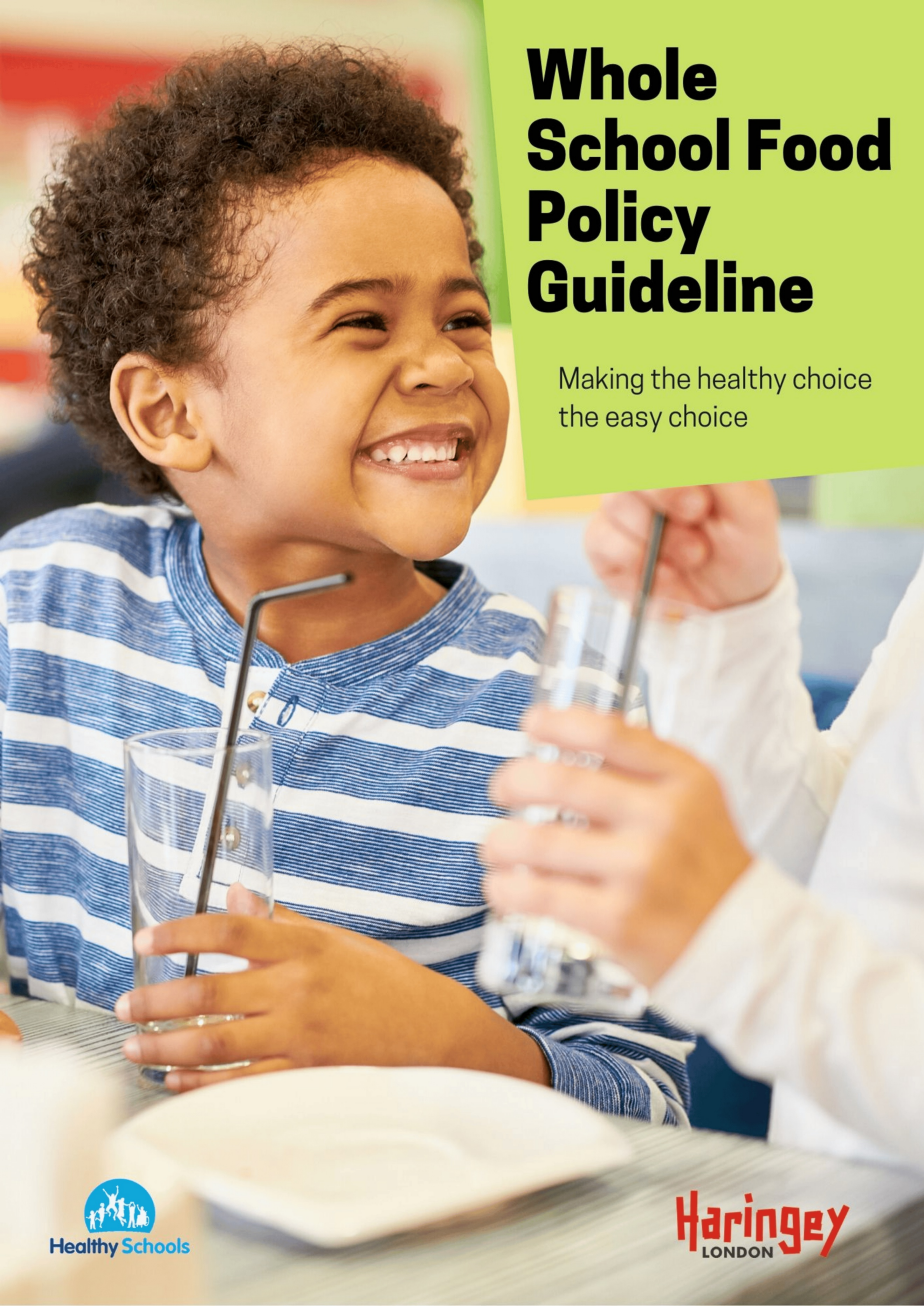
Front page of the Whole School Food Policy Guideline Credit: The authors and Haringey Council.

**Figure 2:**
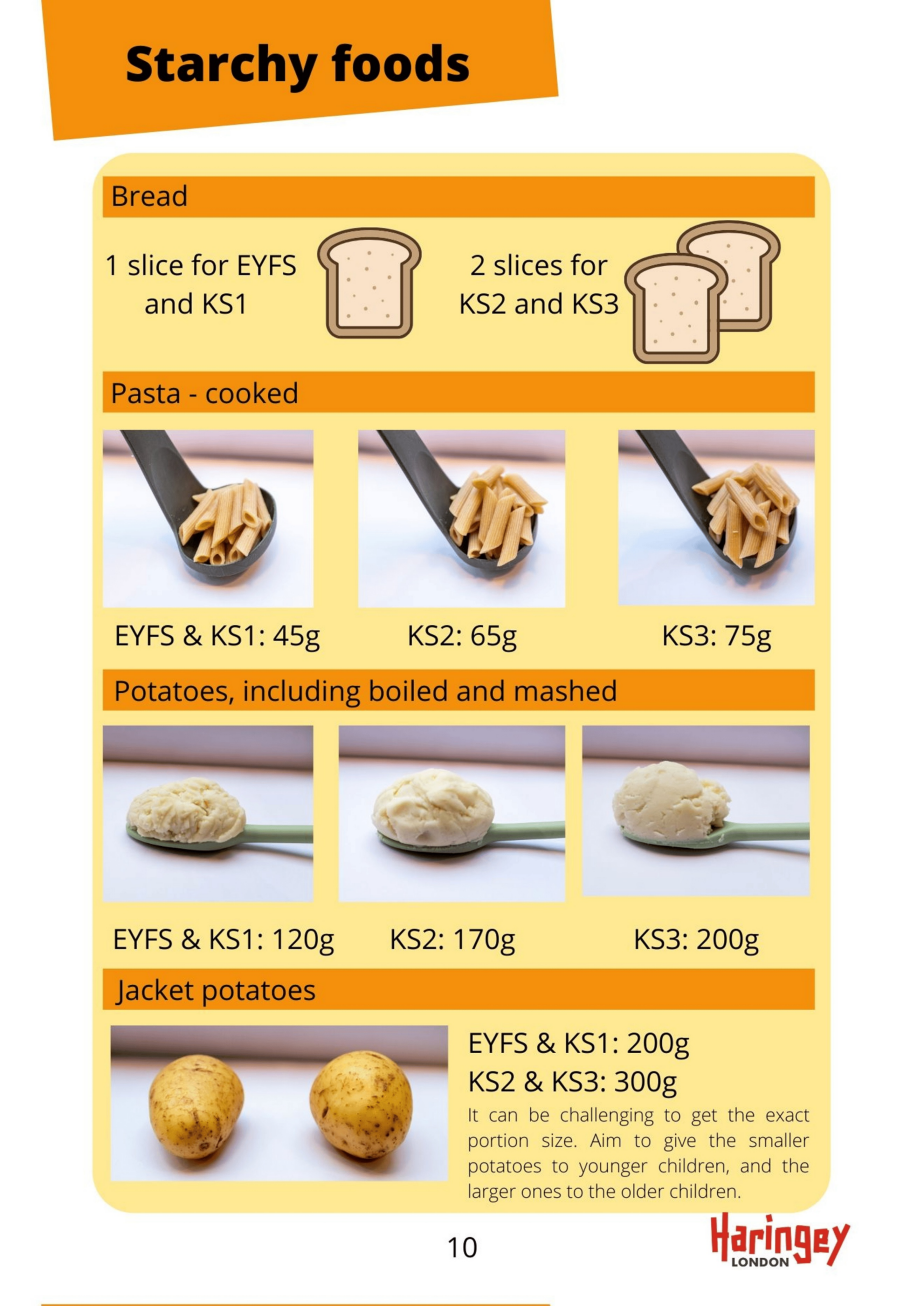
Sample of the portion size guide based on the Enhanced Haringey Food Standards EYFS: Early Years Foundation Stage, KSI: Key Stage 1, KS2: Key Stage 2, KS3: Key Stage 3 Credit: The authors and Haringey Council.

**Figure 3:**
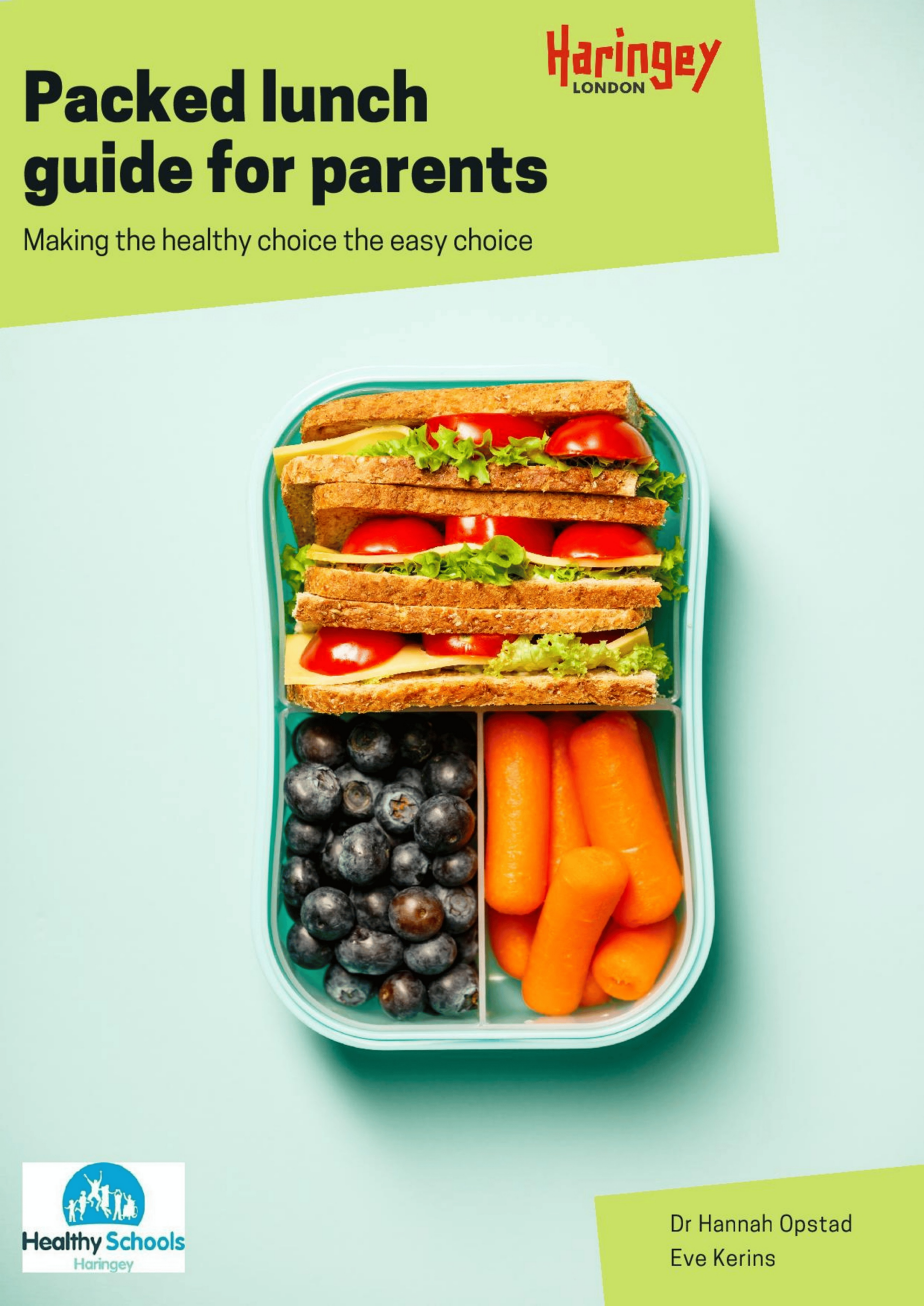
Front page of the Packed Lunch Guide for Parents Credit: The authors and Haringey Council.

**Figure 4:**
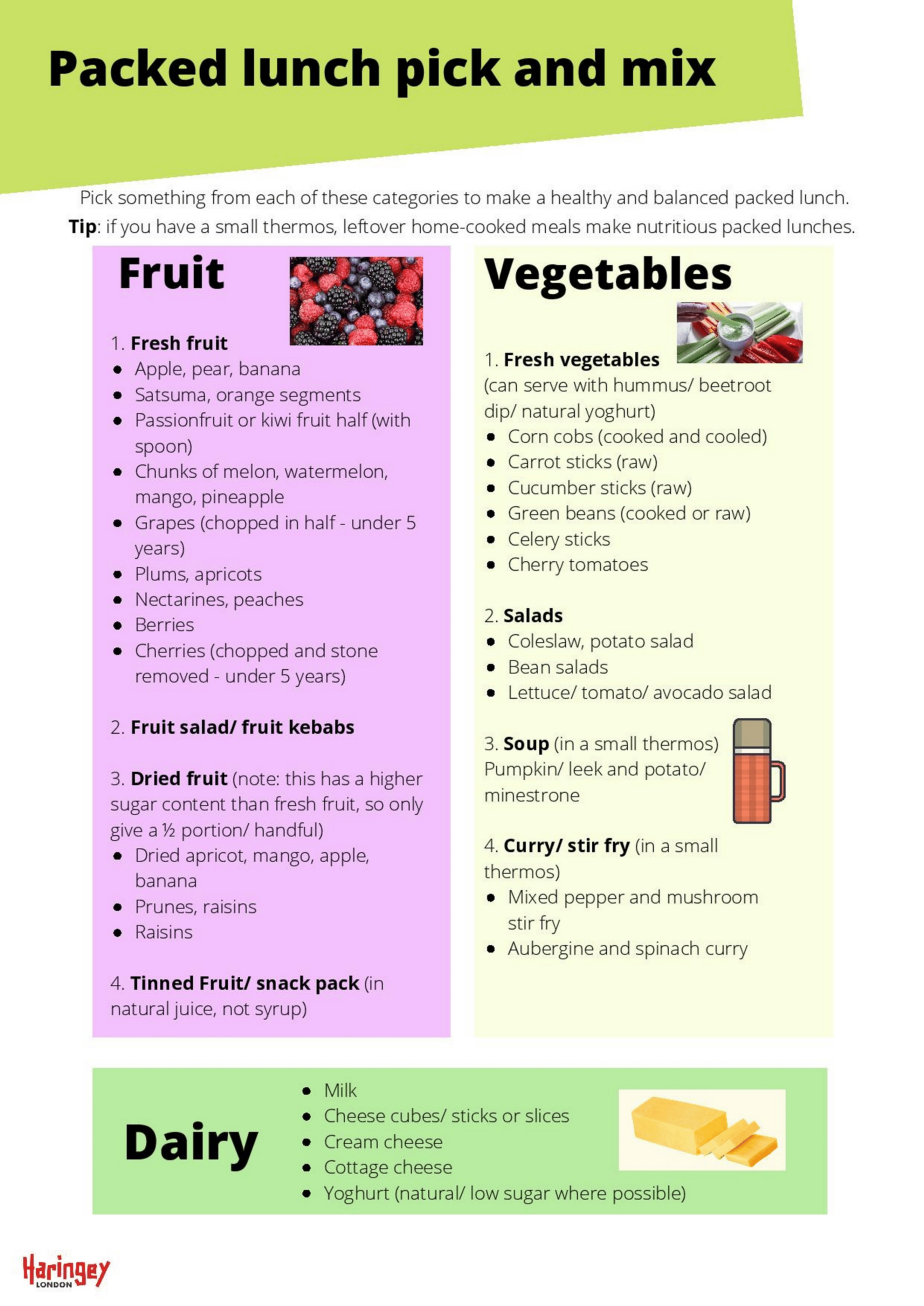
Sample page from the Packed Lunch Pick and Mix Credit: The authors and Haringey Council.

Contents of the guideline can be seen in Table 1, and sample pages from the Guideline can be seen in Figures 1-4.

**Table 1:** Contents of the Whole School Food Policy Guideline

Guideline Contents	
Guidance for helping schools provide healthier lunches	Local food standards were developed with input from dietitians. These go above and beyond the National Food Standards and provide improved and updated recommendations for portions.
	A visual portion size guide, which can be used by kitchen staff to accurately serve age-appropriate portions, from the Early Years to Secondary School.
	Discussion points for schools to follow when procuring new, healthier caterers.
	A Printable Packed Lunch Guide for parents and carers that the school can distribute as needed. This guide includes a "Pick and Mix" section giving examples of each food group and then a visual representation of a balanced meal.
Guidance for developing a healthier food culture	Advice on becoming a water-only school.
	Staff eating with children, providing positive role modelling whilst also quality controlling the food served.
	Upgrading the tuck shop. Providing healthy snacks, getting the students to take responsibility for the shop and providing pre-made healthy dinners for families.
	Creating a space to teach children cooking skills and growing their own food.
	Advice on establishing "nudges", so the school environment naturally promotes healthy choices. This includes placement of healthy snacks at eye-level and using smaller plates to make portions appear bigger.
	Lesson plans to integrate teaching about healthy eating into the National Curriculum.
	Advice on developing School Nutrition Action Groups, where passionate representatives across the school discuss ways to improve the existing food policy.

Participant recruitment

Inclusion criteria were HSLs and staff members in charge of healthy initiatives from all schools who received the guidelines. All schools were invited to interview via email, and those attending a Healthy Schools meeting were invited again. Four interviews were arranged following this.

The interview topic guide was newly formulated during this project, designed to fully address the project’s aims, whilst considering the short time period between guideline release and interviews (see Appendices). The topic guide consisted of five main areas: previous healthy changes, perceived need for the guideline, guideline's impact on opinions, guideline’s recommended healthy changes, and guideline improvements and future resources.

Data collection and analysis

Semi-structured interviews via telephone were chosen as the best form of data collection, in keeping with COVID-19 social distancing guidance in place at the time. Audio recordings were transcribed verbatim. The entire data set was systematically coded using NVivo version 12 software (QSR International Pty Ltd., Melbourne, Australia). Related codes were collated into themes alongside relevant quotes. This process was reviewed and validated by both researchers.

## Results

Interviews were held with three HSLs, referred to as Identity (ID) 1, 2 and 3. This included two mainstream primary schools and one special educational needs (SEN) secondary school. The fourth participant who agreed to be interviewed withdrew due to the COVID-19 school shutdown. One participant sent a follow-up email, which was included in the data analysis.

Five main themes emerged during analysis, labelled: "utility", "facilitators", "barriers", "special schools" and "guideline improvements and future resources". These have been detailed in Table 2.

**Table 2:** Results of thematic analysis

Theme	Subtheme	Quotes	Interpretation
Utility	1.1 Resource quality: relevance and accessibility	“It’s easy to read, it’s very well laid out, it’s lovely and colourful, it’s really attractive… you want to read it.” ID 3 “There’s quite a few (ideas) that… are very realistic and could be done… water-only, the one… when the lunchtime staff save the meal for the end of the day and parents could maybe take it home” ID 2 “I think most of it (the guideline) is appropriate for (our school)… The info on packed lunches and 'water only' school are also appropriate. for (sic) many of our students.” ID 1, follow-up email	The colourful appearance of the guideline engaged readers. The ideas for healthy changes were considered realistic, including within SEN schools.
	1.2 Educational tool	“Parts of it need to be shared with the parents, parts of it need to be shared with the staff, parts of it need to be shared with the children, and the kitchen as well” ID 3	It was found that the guideline could be used as a tool to educate other stakeholders. It clearly illustrates the statistics on obesity rates and gives practical changes that could be implemented.
	1.3 Packed lunch guide	“I was absolutely aghast at what the children are bringing into school. I mean some of them, they’re having… a three-course meal” ID 3 “I really like… the little packed lunch leaflet that comes with (for) parents… it’s nice and colourful, it’s easy to understand and it’s just giving some guidance, isn’t it. It’s not… telling people what they can and cannot do it’s just giving some really good ideas and choices” ID 3 “I did ask for it (packed lunch guide) to be sent to all the parents” ID 3	The packed lunch guide was particularly valued, directly addressing reported concerns about quality of food from home. The only change implemented at point of data collection was one school emailing this guide to parents.
Facilitators	2.1 External professional guidance	“Before that no… there weren’t any official guidelines” ID 3 “I think it’s really informative but it’s not preaching. It’s just setting out the facts in a… very easy to read, friendly way. It’s not pointing the finger, it’s not being judgemental, I like that it’s just dealing in facts” ID 3 “It comes from Haringey… it’s not us, it’s professional people (saying) this is what we should be doing” ID 3 “When you are talking with parents and you are talking with staff, that again it’s about… this is where we’re coming from and this is what our guidelines are… that’s another layer of support isn’t it.” ID 1	The guideline was the first resource of its kind to be offered to schools by the council. The HSLs appreciated that it came from professionals, was factual and came from an organisation external to their environment.
	2.2 Passionate staff	“We’re not a water only school and I’d love to be a water only school” ID 2 “…it’s not just about the academics, really it’s the whole thing. And being healthy is part of that. You know a healthy child is probably a happier one. It’s probably a child who is able to achieve more because they’re nourished. So I think it’s crucial for schools, I really do” ID 3	Passionate staff are key drivers of healthy changes. This includes the interviewees, whose participation in the project reflects their passion for improving student health. The HSLs had passion for healthy initiatives and were striving to establish them.
	2.3 Ideas with memorable titles	“We introduced Wake and Shake which I do in the mornings… it involves music and… five minutes of exercise to get them ready for the day” ID 2 “We did a thing about a year ago and it was WE, ‘we walk for water’, and it’s raising money for water aid… I changed it to ‘we walk, wheel and push for water’” ID 1 “When I saw it about the Teacher Tuesdays, about teachers eating with the children… if we implement things like that it can encourage more children to eat healthy” ID 2	Previously successful changes had catchy, memorable titles, and there was interest in the guideline’s suggestion of ‘Teacher Tuesdays’.
	2.4 Timing of interventions	“But thinking about September… I think we’d really all like to launch off really early. And I say we’ve got some students that are leaving and we’ve got a new cohort of students… so we’ll have new parents” ID 1 “Beginning of the school year so you can say, ‘Yeah you know what, we’re starting afresh this year. We are going to be a water only school” ID 2	Optimising the timing of change was reported as a key consideration to enable success. September was reported as an ideal time, when there is a new parent cohort and the change appears less reactive, potentially reducing resistance from stakeholders.
Barriers	3.1.1 Resistance to change from children	“The children in general they just want to go out and play… a lot of the children say… they don’t like it because there’s, (it’s) probably less sugary and it looks more healthy… obviously in their mind if it looks more healthy they’re not going to like it as much” ID 2	Children are nervous and uninterested in trying new healthy food and there is often no system to engage them, limiting the potential impact of the guideline’s catering recommendations.
	3.1.2 Resistance to change from parents	“When we launched the packed lunch policy, we worked really hard on it… we had a big launch and hardly any parents turned up… I don’t think it’s an easy task to try and change people’s mindsets.” ID 3 “There’s always an uproar… the first time we tried to change and take out snacks… parents weren’t happy” ID 2 “We’ve had parents where we’ve… spoken to them and… some parents get quite defensive” ID 1 “Our parents don’t like to listen. It’s a bit of an uphill struggle if the truth be told” ID 3 “We’ve got some parents where if the children were to go home and say they were being told off about what they’re eating they would be straight into the school complaining” ID 3	A major barrier to the guideline’s impact was parental behaviour. Their resistance to or lack of engagement in change was reported by all HSLs, alongside concerns about the quality of food from home. ID 3 discussed the lack of parental interest in their packed lunch policy. It can be understood that an anticipated backlash would deter schools from instigating the guideline’s recommendations, despite perceived need for change.
	3.1.3 Resistance to change from staff	“She… had a word with the nursery teacher who keeps saying that he talks to the parents (about unhealthy snacks) but we know he doesn’t and we think in a way he kind of turns a blind eye” ID 3 “I need the support of the headteacher as well… she is supportive, but, I think she’s also got a lot of other things” ID 3	Staff may be hesitant to confront parents about the quality of packed lunches. Senior Leadership Teams were key stakeholders to involve but due to the complex nature of their role, did not always prioritise healthy eating.
	3.2 Lack of resources: time, staff, funding	“We did have a no snack policy so they can only bring plain popcorn and… one snack. But… no one’s been on top of it so it’s… reverting back to how it was before” ID 2 “A lot of the things in the guideline would be so easy to implement if we had the money to resource them and we had the staff to monitor it all.” ID 3	High workload prevents staff dedicating time to reviewing the guideline, funding is often required to implement changes and once implemented, recommendations can be unsuccessful if there are insufficient staff to encourage children’s engagement.
Special schools	4.1 Complex needs	“A lot of our students are having major surgery... the dietitian might put them on… supplement drinks if they lose weight… and then conversely we have some students who have to be really careful with their weight because they’re wheelchair users” ID 1 “I mean one of our students… was autistic… he had his set packed lunch and it was set up in this (specific) way... we had staff take food away and of course that didn’t go well” ID 1 “It’s very individualised but I still think that having some structure in place will be good… that you can refer back to” ID 1 “He had one-to-one running round the school with a member of staff… he was doing his Daily Mile” ID 1	The special school reported that they have different baselines for each child’s diet and weight based on their needs. Students with strict routines may struggle with change. The special school’s HSL felt that the guideline would still be useful in their setting despite the complexity of the student’s needs. The special school is resourced to manage these complex needs which was reported to be valuable in implementing individualised plans for children.
	4.2 Support staff	“It’s like the whole school day revolves around snack time which it doesn’t really” ID 1 “We’ve got some support staff who feel that the students are their children… if the students don’t want to (eat), they get very upset and… (they) take it very personally when they’re (the student’s) not eating” ID 1	The relationship between support staff and students was more of a caring role than educational role, such as helping with going to the bathroom and eating. A huge importance was placed on eating within this relationship.
Improvements and future resources	5.1 Water-only policies	“Interviewer: Which changes would you implement first? ID 2: Oh definitely the water only one because… I’ve always wanted to do that and… it’s easier to say… we’re going to become a water only school, and promote it to the children.” “It’s always just been an unwritten rule that you would have never be allowed fizzy drinks and that… really isn’t something that has ever been a problem” ID 3	The guideline’s recommended water only policies were a popular concept, with one school already water only and the other two considering the change. The interviewees felt this would be an uncontroversial change to implement.
	5.2 Outreach to families	“I think the packed lunch guide is really good. But only thing is, I think it should be double-sided and that’s it” ID 2 “They (previous employment) used to do… a Junior Citizens thing in Year 6… they’d have like a card… and they’d have stickers and they had certain jobs they had to do. And then at the end of the year, someone from the MET would come in and… everybody would get a certificate” ID 1 “A cooking… at home guide for children and parents” ID 2 “We do get quite a lot of parents coming (to parent activity days). That would be something else again that you could then lead that onto about healthy snacks and healthy food.” ID 1 “I think things like ‘People Resource’, where people come in and talk to the children…maybe sharing, someone coming in from another school and sharing ideas that they’ve done at their school” ID 3	The usefulness of the packed lunch guide was evident, but both mainstream schools would prefer a double-sided A4 guide. All the information was reported useful but could be split into separate resources simplifying distribution for schools and aiding parents’ engagement. Two interviewees wanted to help parents provide healthier meals. Regarding future resources, they suggested guides and schemes to encourage cooking at home. Another idea mentioned was to have opportunities for education on healthy eating. One suggestion was to host events involving parents, and another was to have sharing of ideas between schools.

## Discussion

Key findings and relation to previous research

A Valued Resource for Haringey’s Needs

This study found that the guideline is an accessible and appropriate resource for Haringey schools. The pleasing layout aids its potential to engage readers and facilitates the communication of its contents. The ideas for healthy changes were considered realistic for the Haringey schools, indicating that the “good contextual fit” criteria for successful initiatives have been met [14]. The HSLs felt it could be used as an educational tool for other stakeholders, and they had plans to share it with their colleagues.

The guideline’s informative tone and authority as a council-produced document were found to take the onus off schools trying to make healthy changes. School food environment policies have been shown to effectively improve students’ dietary intake [7], but previous efforts were met by resistance from both parents and staff, with no formal resource to help. The guideline validates a school’s efforts and condenses a large volume of information into a concise format to support the development of robust policies.

Research has found that effective implementation of healthy initiatives relies on the adaptability of the programme to different settings to enable good contextual fit [15]. This guideline was found to be adaptable for SEN schools; it could be used as a template with alterations made based on the students’ complex needs. However, it did not address the support staff in SEN schools who, in their caring, rather than educational, roles, are key stakeholders to engage in improving healthy eating in these settings.

Outreach to Parents

A major barrier to successful change identified was parental behaviour. Their resistance to change was reported by all HSLs, using words such as “defensive”, “uproar”, “complaining” and “uphill struggle”. This reflects the literature, which finds successful parental engagement in new initiatives challenging and often unsuccessful [15,16]. Adding to this problem, schools were concerned about the quality of food from home. Despite the perceived need for change, an anticipated backlash could deter schools from instigating the guideline’s recommendations.

The packed lunch guide was found to be a particularly valuable resource, possibly as it supports parental engagement; however, the guide should be shorter to aid convenience and accessibility.

Future interventions directly supporting schools to engage parents in healthy changes would be appreciated by the HSLs. Ideas suggested by the HSLs included hosting cooking events with families, running cooking awards schemes or developing more healthy cooking resources such as recipe books.

Hierarchy of Initiatives

This study identified a hierarchy of acceptable healthy initiatives: physical activity, then water and then diet.

Previous physical activity interventions were often successful; stakeholders readily participated with minimal resistance, notably if the intervention had a catchy name. Conversely, dietary interventions were controversial and unsuccessful. A systematic review found that a combination of both physical activity and dietary improvements in school settings results in the most improvement in childhood obesity rates compared to either aspect independently [16]. However, parents feel that they are responsible for their child’s nutrition, with only support required from the school, where, conversely, physical activity is a joint school and parental responsibility [17]. It’s imperative to ensure both physical activity and nutrition are addressed in schools; however, these underlying attitudes must be taken into consideration.

This study went on to find that the guidelines' recommended water-only policies are a novel yet popular concept, with one school already water-only and the other two seriously considering the change. There is little disagreement surrounding the unhealthy nature of sugary drinks, contributing to the easy implementation of water-only policies. Promotion of water-only policies could act as a bridge, shifting stakeholders’ mindsets towards diet-based interventions. Of note, in 2020, the Mayor of London urged all London primary schools to become water-only and developed water-only school toolkits. In 2024, 66.1% of London schools had switched to be water-only schools, with local policies, leadership and resources being key influential factors improving uptake of the water-only school policy [18].

Resources

Haringey schools have free access to the guidelines. Automatic access to this resource, independent of funding or enthusiasm, means that a diverse range of schools can access vital information to improve student health.

However, a lack of resources could limit impact. It was found that a high workload prevents staff from dedicating time to reviewing the guidelines. Funding is then often required to implement changes. Once implemented, recommendations, like improved catering, are unsuccessful if there are insufficient staff to encourage children’s engagement. This could seriously inhibit the guideline’s impact in the local schools.

This was reported to be less of a problem within the SEN school, who have a higher staff-to-student ratio, allowing focused interventions for individual students. However, the guideline hasn’t provided any specific guidance for children with SEN in mainstream schooling, for whom the challenges may be enhanced.

This study found passionate staff are a driving force for change and arguably the most valuable resource. They identify what must change within their own school to improve student health and understand which initiatives would succeed. The presence of passionate staff within schools determines the guideline’s impact as they will prioritise reviewing and instigating its recommendations in an appropriate manner. Future interventions would do well by focusing on the engagement of these motivated members of school staff.

Further research

Further research should focus on the guideline’s usefulness in a wider range of Haringey schools; those in higher socioeconomic areas, secondary schools and more SEN schools, to further explore the interesting themes that emerged. The guideline could then be adapted for different types of schools, primary, secondary and SEN, to more appropriately meet their individual needs.

It would be valuable to investigate the guidelines' impact within Haringey schools over a longer time period than this study. This could be through monitoring obesity rates, measured through the NCMP, or recording how many Haringey schools meet certain targets towards establishing a whole school food policy. Taking a wider perspective, it would be valuable to investigate the effect of Haringey Council’s Whole Systems Approach to tackling obesity - of which this guideline was a single component - on the whole borough’s obesity rates and obesity-related spending.

Lastly, parents are key stakeholders in childhood obesity, but they were found to be a barrier to changes in schools. Holding focus groups with parents could explore their perspectives and identify ways to engage them in new initiatives. In this way, schools could more successfully improve their food policies to benefit student health.

## Conclusions

The findings of this study are positive but must be considered in context. The three participating schools are all engaged with the council in healthy school initiatives. Various barriers to guideline implementation were identified, but not mitigated. The guideline’s impact within the borough was limited by the COVID-19 pandemic, towards which focus had shifted within both the public health and education sectors. Furthermore, the participating schools had two months to engage with the guidelines prior to the interview, and assessment of the long-term impact was beyond this study’s scope.

This study focused on HSLs, only one relevant stakeholder in childhood obesity. It is imperative that future interventions incorporate others, namely, parents. Going through schools could be an effective way of reaching parents, perhaps through coffee mornings and activity days which already take place at some schools. Such efforts could increase the success of healthy changes and have a greater impact on student health. This study identified that schools value outside professional guidance and realistic suggestions for improving student health. However, guideline development was the first step towards promoting whole school food policies in Haringey, and its impact is dependent on integration within current practice. Hopefully, this guideline will support schools in creating healthy food policies that make the healthy choice the easy choice for students.

## Appendices

**Table 3:** Interview topic guide

Topic	Questions
Introduction	What’s your role in the school? What’s your role regarding the health of the students? Have you got a Healthy Schools London award? (not registered/registered/bronze/ silver/gold)
Previous healthy changes	Have you tried to make healthy changes in the school before the guidelines were sent out? What have you changed? How did you find making this change? What motivated you to do this? What guidance did you use for any changes you’ve implemented? (If no), what were the barriers to making healthy changes?
Need for the guideline	Do you think the guidelines are needed by your school? Have you ever seen anything like the guideline before? What do you think is the most important thing your school needs to change to improve the health of your students? Will this guideline help to achieve this change?
Impact on opinions	Childhood obesity is an increasing problem in the UK at the moment. How much of an issue do you think this is in your school? How has your opinion on this been impacted by the guideline? What do you think about the roles of schools in promoting children’s health? How has your opinion on this been impacted by the guideline?
Healthy changes	What did you think about the guideline? What did you think about the ideas for healthy changes covered by the guideline? Were the ideas realistic for your school? Has your school implemented any of these healthy changes since receiving the guidelines? Are you planning to make any changes? (For schools planning on making changes), which changes are you planning to implement first? Why? What are the barriers to making other changes?
Guideline improvements	What do you think could be improved about the guideline? What other resources would help your school make healthy changes?
